# Supplementary material for: Multiple anthropogenic pressures eliminate the effects of soil microbial diversity on ecosystem functions in experimental microcosms
Source: Nat Commun. 2022 Jul 23;13:4260. doi: 10.1038/s41467-022-31936-7 (PMC9308766; doi:10.1038/s41467-022-31936-7)
Supplement: Supplementary file 1 — Supplementary Information [file 41467_2022_31936_MOESM1_ESM.pdf]

Multiple anthropogenic pressures eliminate the effects of soil microbial diversity on ecosystem functions in experimental microcosms

Gaowen Yang[1,2,3*], Masahiro Ryo[4,5], Julien Roy[2,3], Daniel R. Lammel[2,3], Max-Bernhard Ballhausen[2,3], Xin Jing[6], Xuefeng Zhu[7], Matthias C. Rillig[2,3]

[1] College of Grassland Science and Technology, China Agricultural University, 100193 Beijing, China

[2] Institute of Biology, Freie Universität Berlin, 14195 Berlin, Germany

[3] Berlin-Brandenburg Institute of Advanced Biodiversity Research (BBIB), 14195 Berlin, Germany

[4] Leibniz Centre for Agricultural Landscape Research (ZALF), 15374 Müncheberg, Germany

[5] Institute of Environmental Sciences, Brandenburg University of Technology Cottbus-Senftenberg (BTU-CS), 03046 Cottbus, Germany

[6] State Key Laboratory of Grassland Agro-Ecosystems, and College of Pastoral Agriculture Science and Technology, Lanzhou University, 730020 Lanzhou, Gansu, China

[7] Institute of Applied Ecology, Chinese Academy of Sciences, 110016 Shenyang, China

Correspondence and requests for materials should be addressed to G.Y. (email: yanggw@cau.edu.cn)

Supplementary Information

Supplementary information provides the methodology in detail, and explains how indices were calculated, and how they were statistically analyzed and visualized. This file includes:

Supplementary Methods

Supplementary Table 1

Supplementary Fig. 1 - 14

Supplementary Methods

Manipulation of soil microbial diversity

We collected field soil from the top 10 cm of a farmland in Albrecht-Thaer-Weg, Berlin (52.466°N, 13.303°E). The soil is an Albic Luvisol and has the following properties: 73.6% sand, 18.8% silt and 7.6% clay; pH 7.1 ($CaCl_2$), 6.9 mg P/100 g soil (calciumacetate-lactate), 0.12% nitrogen and 1.87% carbon[1]. Field soil was passed through a 2 mm mesh to remove large roots and stones. We sterilized 20 kg of soil for 90 min at 121°C, and stored 2 kg of fresh soil at 4°C.

We used the dilution-to-extinction approach[2-6] to create the high and low soil biodiversity (Supplementary Fig. 1). During the dilution, the loss of rare species occurs first, followed by more abundant species. This method can create soil communities with a realistic gradient of species richness, because rare soil microbial species are likely more sensitive to GCFs[7,8]. We prepared a parent inoculum suspension by mixing 100 g of fresh soil in 200 ml of sterilized VE water. The mixture was vortexed at high speed for 5 min to mix the contents. The sediment settled for 1 min. The upper 200 ml of soil suspension was treated as parent inoculum suspension. 50 ml of parent inoculum suspension was added to 500 g of sterilized soil in a plastic bag, and homogenized by turning the bag up and down for 30 times to obtain the inoculum of high soil biodiversity. Another 5 ml of parent inoculum suspension was mixed with 45 ml of sterilized parent inoculum suspension to create the 10^{-1} dilution. This procedure was repeated for five times to reach the 10^{-6} dilution. 50 ml of the 10^{-6} dilution was mixed and homogenized with 500 g of sterilized soil in a plastic bag to obtain the inoculum of low soil biodiversity. There were 10 bags (five bags for each soil biodiversity treatment) in total.

Sterile water was added to each plastic bag to reach the water content of the fresh field soil. All bags were closed with a sterilized cotton plug and a rubber band to avoid microbial contamination but allow gas exchange[6]. All bags were incubated in a dark room (20 °C) until similar microbial abundance was observed among dilution treatments. The soil in each bag was homogenized by shaking and turning the bags every week. Quantitative real-time PCR (qPCR) was employed to determine fungal and bacterial abundance[3]. The details of qPCR have been described in *the measurement of response variables*. In the present study, it took two months to recover soil microbial biomass (Supplementary Fig. 3).

Global change factors

The pool of 10 CGFs included: warming (+ 5.0 °C), nitrogen deposition (100 kg N ha^{-1} yr^{-1}), drought (30% of soil water holding capacity compared to 60%), heavy metal pollution (100 mg Cu kg^{-1} as $CuSO_4$), film residues (4.0 g polyethylene films kg^{-1} soil), salinity (4.0 dS m^{-1} with NaCl), agricultural fungicide application (125.0 mg Captan kg^{-1} soil), agricultural bactericide application (10.0 mg Bronopol kg^{-1} soil), surfactant contaminant (1.0 g sodium dodecylbenzenesulfonate kg^{-1} soil), soil compaction (1.7 g cm^{-3} compared to 1.3 g cm^{-3}). Agroecosystem, one of most intensively managed ecosystem, is affected by these multiple GCFs at a time. Our study focused on these GCFs, which frequently occur in agroecosystem. We here present the rationale for the 10 tested GCFs.

1) Warming (+5.0 °C). We used 20°C as the ambient temperature, because it is the average soil temperature (0-10 cm) from May to September during the past three years in the sampling site. An increment of 5.0 °C over the ambient temperature is frequently used in experiments testing the effect of warming, and this increment is the predicted climate scenario for the next 100 years[9,10].

2) Nitrogen deposition (100 kg N ha^{-1} yr^{-1}). Nitrogen (N) enrichment from N fertilizer or atmospheric N deposition has been recognized as an important GCF[11]. We added N at a high N enrichment rate as NH_4NO_3 at 100 kg N ha^{-1} yr^{-1} [10]. Based on the soil surface area of each experiment unit, we added 5.72 mg N (equal to 16.34 mg NH_4NO_3) to each experiment unit with N enrichment treatment.

3) Drought. The frequency and intensity of drought events have been predicted to increase in many regions[12,13] and strongly affect global crop yields[14]. We used 60% of water holding capacity as the control water level, and 30% as the drought treatment. This level of drought has been widely used in the previous studies[10,15,16].

4) Heavy metal pollution. Heavy metal, non-degradable inorganic compounds, can come from mining, atmospheric deposition, wastewater, and organic feralization, leading to contamination in agroecosystem. There is growing concern that the accumulation of heavy metals in soil poses high risks to agroecosystem and human health[17,18]. We used copper to represent heavy metal in this study, because it is an important soil pollutant in Europe[19]. Copper was added as $CuSO_4$ at the rate of 100 mg Cu kg^{-1} soil to simulate a hotspot of copper contamination[10].

5) Film residues. The residual plastic film from the intensive use of plastic mulching has been shown to cause pollution hazards to agroecosystem[20,21]. In this study, plastic mulch films (polyethylene) were cut into rectangular pieces with side lengths ranging from 4 mm to 10 mm and were added at a concentration of 0.4% (w/w) of soil[21].

6) Salinity. Soil salinization, a worldwide threat to soil degradation, has been recognized as a major human-induced GCF[22-25]. We used soils with an electrical conductivity of 4.0 dS m^{-1} as salinity treatment. This level of salinity is regarded as moderately saline[10]. In this study, we added NaCl at the rate of 472 mg NaCl/ 40 g sterilized soil (dry weight) to achieve the electrical conductivity of 4.0 dS m^{-1}. This amount of NaCl was achieved by a preliminary experiment. First, 100 ml of VE water was mixed with 20 g (dry weight, d.w.) of sterilized soil, was shaken for 1h, and then the electrical conductivity was determined using SevenEasy pH Meter (Mettler-Toledo GmbH, Giessen, Germany). Second, NaCl was gradually added to reach 4.0 dS m^{-1} and it was found that 236 mg NaCl is needed for 20 g (d.w.) of sterilized soil.

7) Agricultural fungicide application. Recent studies observed the widespread occurrence of pesticides in agricultural soils, which can be detrimental to soil functions and organisms[26,27]. Captan is one of the most commonly used fungicides worldwide in farmland. We used a recommended dose of Captan (PESTANAL ® analytical standard, Sigma-Aldrich, MO, USA, catalog #32054) at 125.0 mg kg^{-1} soil as agricultural fungicide treatment[28].

8) Agricultural bactericide application. Bronopol is a widely used bactericide. Previous studies showed that the addition rate of above 10.0 mg kg^{-1} soil was used to suppress soil bacteria[29-31]. Here, we used 10.0 mg Bronopol (PESTANAL ® analytical standard, Sigma-Aldrich, MO, USA, catalog #32053) kg^{-1} soil as agricultural bactericide treatment.

9) *Surfactant contaminant*. Surfactant, as a surface active agent, can enter agroecosystem along with pesticide and organic fertilizer applications. Sodium dodecylbenzenesulfonate is the most widely used surfactant, ranging from 11 - 30,000 mg kg^{-1} in sewage sludge application[32]. We add sodium dodecylbenzenesulfonate (Sigma-Aldrich, MO, USA, catalog #STBH6698) at 0.1% (1000 mg/kg).

10) Soil *compaction*. The intensification of mechanical operations exacerbated soil compaction in agroecosystem, which has a profound harmful effect on soil abiotic and

biotic properties[33-35]. Soil bulk density between 1.6-1.8 g cm^{-3} was classified as medium harmful soil compaction[36]. In this study, 1.7 g cm^{-3} of soil bulk density was used in the compacted treatment. This level of bulk density was found to occur following mechanical operations at the sampling site. The average soil bulk density was 1.3 g cm^{-3} in the farmland where we collected soils, which was treated as the uncompacted control treatment.

The measurement of response variables

We measured the following response variables: microbial activity (soil respiration), microbial abundance (bacterial and fungal abundance), nutrient cycling (litter decomposition rate and soil enzyme activity), physical properties (water-stable soil aggregates and soil water repellency), bacterial and fungal biodiversity and community composition.

1) ***Soil respiration***. Soil respiration is often used as an indicator of soil microbial activity. We measured soil respiration as CO_2 concentration (ppm h^{-1} g^{-1} soil) in the third and sixth week. Mini Bioreactors were opened for 5 min, and then were sealed with sterilized lips (70% alcohol for 10 min) and incubated for about 2 hours. Each lid was modified to contain a septum that allowed sampling of CO_2. We sampled 1 ml of air from the headspace of each Mini Bioreactor using a medical syringe at the beginning of incubation and recorded the starting time. About 2 hours later, another 1 ml of air from each Mini Bioreactor was sampled, and the ending time was recorded. Air samples were injected into an infrared gas analyzer (LiCOR 6400xt, Lincoln, NE, USA) to measure CO_2 concentration. Soil respiration was determined by subtracting the CO_2 concentration at the beginning of incubation from that at the ending time. After soil respiration determination, sealed lips were replaced by the previous caps with vents.

2) **B*acterial abundance***. Bacterial abundance was estimated by quantitative real-time PCR (qPCR) targeting the 16S rRNA gene. (a) *Standard preparation*. First, qPCR standards were prepared by pooling all DNA extractions at equal volumes (1 µl for each sample). Then we used this mixture as a template for PCR amplification with the primers 27F and 1492R[37]. Amplification was performed using a Biometra Tone thermocycler (Analytik Jena, Jena, Germany) in 50 µl reaction mix: 10.0 µl of 5x KAPA HiFi Fid buffer with Mg, 1.5 µl of 10 mM KAPA dNTP Mix, 1.0 µl of 10 µM of each primer, 0.5 µl of 1 U μl^{-1} of KAPA HiFi polymerase (Kapa Biosystems, Woburn, MA, USA), 1 µl of DNA template,

and 35.0 µl PCR-grade water. The PCR cycle is as follows: a denaturation step for 3 min at 95°C, 30 cycles of denaturation for 20 s at 95°C, annealing for 30 s at 58°C, and elongation for 1.5 min at 68°C, and a final elongation step of 5 min at 68°C. We examined PCR products on a 1.0% agarose gel using electrophoresis (the expected amplicon size is 1465 bp) and subsequently purified PCR magnetic beads (NucleoMag NGS Clean-up and Size Selection, MACHEREY-NAGEL GmbH & Co.KG, Düren, Germany) in a 0.8:1 ratio (bead:PCR product), following the manufacturer's instructions. The DNA concentration of the PCR products was measured using Qubit 3 Fluorometer, following the manufacturer's instructions. We diluted the PCR product first to 1×10^{10} copies µl^{-1} and then diluted it to produce a standard curve with the concentrations: 1×10^{7}, 1×10^{6}, 1×10^{5}, 1×10^{4}, 1×10^{3}, and 1×10^{2} copies µl^{-1}.

(b) *Sample measurement*. For the qPCR, we used a CFX Real-Time System (C1000 Touch™ Thermal Cycler, BIO-RAD, California, USA) with the specified 96-well white plates (Hard-Shell PCR Plates, Catalog #HSP9655, Bio-Rad Laboratories, Inc., California, USA). Each plate was set, including the standards (tree replicates of each dilution), blanks/negative controls (at least three) and the samples of soil DNA extraction (at least two technical replicates). The qPCR was conducted in 20 µl reaction mix: 4.0 µl of 5x KAPA HiFi Fid buffer with Mg, 0.6 µl of 10 mM KAPA dNTP Mix, 0.5 µl of 10 µM of each primer (515f-y/806r – widely used primers for bacteria and the same that we used for sequencing)[38], 0.2 µl of 1 U µl^{-1} of KAPA HiFi polymerase, 5 µl of DNA template (dilute it 1:5 before using), and 9.1 µl PCR-grade water, and 0.1 µl of 100 µM EvaGreen Fluorescent DNA stain. The qPCR cycle was set as follows: a denaturation step for 3 min at 95°C, 40 cycles of denaturation for 20 s at 95°C, annealing for 30 s at 50°C, and elongation for 30 s at 68°C, and a final elongation step of 5 min at 68°C. A denaturation curve was set at the end of the cycles to confirm the product specificity. The data were analyzed by the Bio-Rad CFX Maestro 1.0 software (Bio-Rad Laboratories, Inc., California, USA), and the copy number data of the samples were exported for further analysis. Some qPCR products were examined by electrophoresis and confirmed the denaturation curve results and the specificity of the amplification. No amplification was observed in the blanks/negative controls.

3) ***Fungal abundance***. Fungal abundance was estimated by qPCR targeting the fungal ITS region. (a) *Standard preparation*. The pooled DNA extraction (described above) was also used as a template for the standard preparation. DNA template was amplified with the primers 5.8s and ITS1f[38] using Biometra Tone (Analytik Jena, Jena, Germany) in a 25 µl volume containing 0.5 µl of 1 U μl^{-1} of KAPA HiFi polymerase, 5.0 µl of 1x KAPA HiFi buffer, 0.75 µl of 10 mM of dNTP, 0.5 µl of 10 µM of each primer, 2 µl of DNA template and 15.75 µl PCR-grade water. The PCR cycles were as follows: a denaturation step for 3 min at 95°C, 30 cycles of denaturation for 20 s at 98°C, annealing for 30 s at 65°C, and elongation for 30 s at 72°C, and a final elongation step of 2 min at 72°C. We examined PCR products on a 1.0% agarose gel using electrophoresis (the expected amplicon size is about 300 bp) and subsequently purified PCR magnetic beads (see above) in a 0.8:1 ratio (bead:PCR product). The DNA concentration of the PCR products was measured using Qubit 3 Fluorometer, following the manufacturer's instructions. We diluted the PCR product first to 1×10^{10} copies μl^{-1} and then diluted it to 1×10^7, 1×10^6, 1×10^5, 1×10^4, 1×10^3, and 1×10^2 copies μl^{-1} as standards.

(b) *Sample measurement*. Quantitative real-time PCR was conducted as described above using CFX Real-Time System (C1000 Touch™ Thermal Cycler, BIO-RAD, California, USA) in 25 µl reaction: 12.5 µl of Mastermix (PowerUp™ SYBR™ Green Master Mix, Thermo Fisher Scientific, MA, USA), 1.25 µl of 10 µM of each primer (5.8s and ITS1f), 10 µl of DNA template (dilute it 1:5 before using). The PCR was performed as follows: a UDG activation for 2 min at 50°C, a denaturation step for 2 min at 95°C, 40 cycles of denaturation for 15 s at 95°C, annealing for 15 s at 53°C, and elongation for 15 s at 72°C. Bio-Rad CFX Maestro 1.0 software (Bio-Rad Laboratories, Inc., California, USA) was used for streamlining plate setup and collection of fungal and bacterial qPCR data. Copy number (log transformed) was used as fungal and bacterial abundance indicators.

4) ***Decomposition rate***. We weighed about 100 mg (d.w.) of *Medicago lupulina* leaves (65°C for 72 h) and then placed them into a 1.5 × 3.0-cm mesh bag with a pore size of 25 µm. Each Mini Bioreactor contained a mesh bag buried 1 cm below the soil surface. After the final harvest, all mesh bags were oven-dried at 65°C for 72 h and weighed. The proportional loss of dry litter weight during soil incubation was used as an indication of decomposition rate.

5) ***Activity of four soil enzymes***. We measured the activity of β-glucosidase (cellulose degradation), β-D-celluliosidase (cellulose degradation), N-acetyl-β-glucosaminidase (chitin degradation) and phosphatase (organic phosphorus mineralization) using high throughput microplate assay[39,40]. We prepared the corresponding p-nitrophenyl (*p*NP)-linked substrate solutions: 5 mM *p*NP-β-D-glucopyranoside (Sigma-Aldrich, MO, USA, Sigma no. N7006), 2 mM *p*NP-β-D-cellobioside (Sigma no. N5759), 2 mM *p*NP-N-acetyl-β-D-glucosaminide (Sigma no. N9376) and 5 mM *p*NP- phosphate disodium salt hexahydrate (Sigma no. 71768), separately. About 5.0 g of fresh soil samples were mixed with 8.0 ml of 50 mM sodium acetate buffer (pH 5.0-5.5). For each reaction of each enzyme, 150 μl of soil suspension was mixed with 150 μl of substrate solution. Reaction mixtures placed in 96 deep-well microplates were incubated in the dark at 20°C for 2 - 4 h. After incubation, we centrifuged microplates at 3,000 rpm for 5 min, pelleted 100 μl of suspension into a new microplate, and stopped the reaction by adding 200 μl of 0.1 M NaOH. We measured absorbances spectrophotometrically at 410 nm using a microplate reader (BioRad, Benchmark Plus, Japan). Absorbances were converted to *p*NP concentration using a standard curve. Enzyme activity was calculated based on the released *p*NP (μmol) per gram of dry soil per hour.

6) ***Water-stable soil aggregates***. A modified protocol by Kemper and Rosenau was used to measure water-stable soil aggregates[41]. We placed 4.0 g of each soil sample on sieves with a mesh size of 0.25 mm, used capillary re-wetting with deionized water for 5 min, and subsequently inserted samples into a sieving machine (Agrisearch Equipment, Eijkelkamp, Giesbeek, Netherlands) and ran the machine for 3 min. The fractions left on the sieves containing sand particles and organic debris were oven-dried for 24h at 60°C and weighed. The percentage of water-stable soil aggregates was calculated after excluding sand particles and organic debris larger than 0.25 mm.

7) ***Soil water repellency***. Soil water repellency was measured using the water drop penetration time method[42]. We placed a droplet (8 μl) of VE water onto the soil surface. The time was recorded until the droplet soaks into the soil. Each sample was carried out in triplicate.

8) ***Bacterial and fungal diversity***. High throughput sequencing (Illumina MiSeq) was used to measure the taxonomic composition of soil fungal and bacterial communities. We used

the primers fITS7 and ITS4 for fungi and 515f-y and 806r for bacteria[38,43]. The PCRs for bacteria were performed in a 25 μl volume containing 0.50 μl of 1 U $μl^{-1}$ of KAPA HiFi polymerase (Kapa Biosystems, Woburn, MA, USA), 5.0 μl of 5x KAPA HiFi buffer, 0.75 μl of 10 mM of dNTP, 0.75 μl of 10 μM of each primer, 10 μl of DNA template and 7.25 μl PCR-grade water. The PCR cycles for bacteria were as follows: a denaturation step for 3 min at 95°C, 30 cycles of denaturation for 30 s at 98°C, annealing for 30 s at 55°C, and elongation for 30 s at 72°C, and a final elongation step of 5 min at 72°C.

The PCRs for fungi were performed in a 25 μl volume containing 0.50 μl of 1 U $μl^{-1}$ of KAPA HiFi polymerase (Kapa Biosystems, Woburn, MA, USA), 5.0 μl of 5x KAPA HiFi buffer, 0.75 μl of 10 mM of dNTP, 0.75 μl of 10 μM of each primer, 8 μl of DNA template and 9.25 μl PCR-grade water. The PCR cycles for fungi were as follows: a denaturation step for 3 min at 95°C, 30 cycles of denaturation for 20 s at 98°C, annealing for 20 s at 50°C, and elongation for 30 s at 72°C, and a final elongation step of 5 min at 72°C.

Both bacterial and fungal PCR was performed in duplicates. PCR products were examined on a 1.0% agarose gel using electrophoresis. The expected amplicon size is about 250 bp for bacteria and 300 bp for fungi. We purified PCR products using magnetic beads (see above) in a 0.8:1 ratio (bead:PCR product). The purified products were used in an indexing PCR with sample-specific primers containing the sequencing adaptors and a 8 nt long index sequence for multiplex sequencing. Indexing PCRs for bacteria fungi were performed in a 25 μl volume containing 0.50 μl of 1 U $μl^{-1}$ of KAPA HiFi polymerase (Kapa Biosystems, Woburn, MA, USA), 5.0 μl of 5x KAPA HiFi buffer, 0.75 μl of 10 mM of dNTP, 3.0 μl of 2.5 μM of each specific primer, 4 μl of DNA template and 8.75 μl PCR-grade water. The indexing PCR cycles for bacteria and fungi followed the similar cycle set up as described for PCR cycles. We again purified indexing PCR products with magnetic beads in a 0.8:1 ratio (bead:PCR product).

Purified indexing PCR products were examined on a 1.0% agarose gel using electrophoresis. The expected amplicon size is about 350 bp for bacteria and 400 bp for fungi. DNA concentration of each purified indexing PCR product was measured using a Picogreen assay as described above for the determination of soil DNA extraction. The final PCR products were pooled on an equimolar basis to obtain the amplicon library. Tapestation D-1000 (Agilent, Waldbronn, Germany) and a D1000 Screen Tape were used

to confirm that primers were absent in the amplicon library[44]. The DNA concentration of the amplicon library was measured using Qubit 3 Fluorometer, and then the amplicon library was diluted to a concentration of 5 ng/µl and sequenced on an Illumina MiSeq platform (Illumina Inc., San Diego, CA, USA) at the Berlin Center for Genomics in Biodiversity Research (BeGenDiv, Berlin, Germany) using v3 chemistry (2x300-bp paired-end sequencing).

We used DADA2 in R to obtain denoised, chimera-free, non-singleton fungal amplicon sequence variants (ASVs)[45], following standard operating procedure[46]. Raw reads were demultiplexed, allowing no error in the index sequence for sample assignment. Primers were removed, and, for fungi, this included the removal of the reverse complement sequence of the reverse and forward primer sequence in the forward and reverse reads, respectively, using cutadapt[47]. Reads with more than 1 and 2 maximum expected number of errors for forward and reverse reads were excluded. Non-singleton ASVs were inferred on a sample basis. Chimera was identified de novo as sequences that corresponded to subsets of two more abundant sequences and removed. Taxonomic annotation of fungal ASVs was performed using the Naive Bayesian Classifier[48] against UNITE[49]. The Silva 138.1 database was used for bacteria taxonomic annotation[50,51]. Taxonomic annotations at any rank were considered robust at a 100% bootstrap confidence threshold. ITS ASVs and 16S ASVs not annotated to fungi and bacteria, respectively, were removed. Sample reads were rarefied to 2000 reads for bacteria and 1000 reads for fungi to account for varying sequencing depth among samples (Supplementary Fig. 12). Number of observed ASVs were treated as an index of bacterial and fungal diversity. The relative abundance was used to visualize the taxonomic composition of bacterial and fungal communities (Supplementary Fig. 8 and 13).

Cohesion calculation

To further investigate microbial interactions and quantify the connectivity of microbial communities, we calculated cohesion values, following Herren and McMahon (2017)[52]. Cohesion value provides insights into associations among microbial taxa caused by both positive and negative species interactions. The cohesion value represents the strength and number of positive and negative associations. Microbial communities with greater negative cohesion and less positive cohesion are more stable[53]. The results of cohesion analysis

indicated that neither the increasing number of GCFs nor soil biodiversity treatment caused consistent changes in positive and negative cohesion (Supplementary Fig. 14).

Quantifying ecosystem multifunctionality

We adopted an averaging approach to quantify ecosystem multifunctionality[54] using eight out of 16 response variables (see the above section for the corresponding functions/properties represented by each response variable). These response variables were soil respiration, litter decomposition rate, the activity of β-glucosidase (cellulose degradation), β-D-celluliosidase (cellulose degradation), N-acetyl-β-glucosaminidase (chitin degradation) and phosphatase (organic phosphorus mineralization), and water-stable soil aggregates. We standardized all response variables to the same comparison scale using the z-score transformation. Before the standardization, soil respiration was reflected using the form of $-fi + \max(fi)$[55], because a low value of soil respiration is considered as a desirable ecosystem function. We calculated an unweighted multifunctionality metric and a weighted multifunctionality metric. The former was the mean values of the eight standardized response variables. The latter was calculated using the methods presented by Manning *et al.* (2018)[56]. Specifically, a cluster analysis was conducted to produce a dendrogram tree using hierarchical agglomerative clustering. The optimal number of clusters was determined using the Elbow method[57]. Individual functions were weighted based on five clusters (i.e. 0.5 for the activity of β-glucosidase and β-D-celluliosidase, 0.5 for litter decomposition rate and N-acetyl-β-glucosaminidase, 0.5 for the first and the last respiration, and 1 for the remaining response variables). The weighted multifunctionality metric was calculated by taking the mean values of the all the weighted individual response variables. We reported results for the weighted multifunctionality metric because it improved the normality of data distribution.

Supplementary Table 1 Effects of soil biodiversity and the number of GCFs on the community composition of fungi and bacteria using the permutational multivariate analysis (ADONIS) of variance based on the Bray-Curtis distance. The statistical test used was two-sided.

Bacterial community composition				
Factors	df.	*F* value	R^2	P value
Soil biodiversity (SB)	6	3.919	0.09553	< 0.001
Number of GCFs	1	51.28	0.20835	< 0.001
SB × GCF	6	2.723	0.06637	< 0.001
Residuals	155		0.62975	
Fungal community composition				
Factors	df.	*F* value	R^2	P value
Soil biodiversity (SB)	6	4.391	0.10654	< 0.001
Number of GCFs	1	46.411	0.18769	< 0.001
SB × GCF	6	2.42	0.05871	< 0.001
Residuals	160		0.64706	

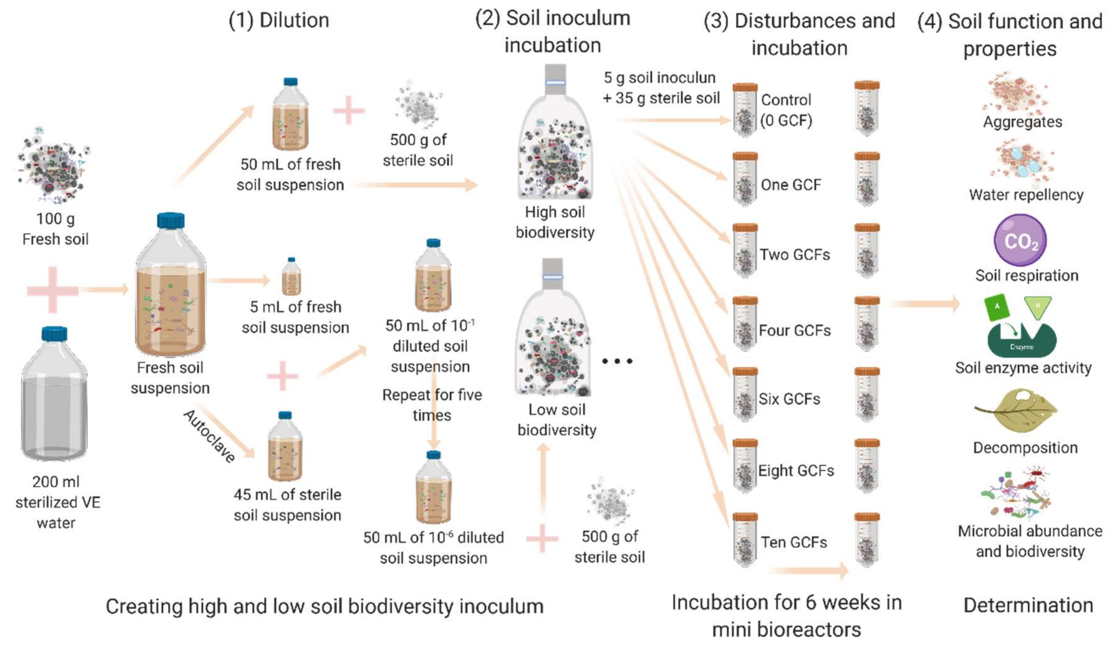

Supplementary Fig. 1 Scheme of the experimental setup and measurement. **(1) Dilution**. The fresh soil suspension was prepared by mixing 100 g of fresh field soil with 200 ml of sterilized VE water. Half of the fresh soil suspension was autoclaved at 121℃ for 20 min to produce sterile soil suspension. We mixed 50 ml of fresh soil suspension with 500 g of sterile soil to obtain a high soil biodiversity treatment. We mixed 5 ml of fresh soil suspension with 45 ml of sterile soil suspension to obtain 10^{-1} diluted soil suspension. This procedure was repeated five times to reach 10^{-6} diluted soil suspension. The low soil biodiversity treatment was created by mixing 50 ml of 10^{-6} diluted soil suspension with 500 g of sterile soil to obtain a. **(2) Soil inoculum incubation**. Each mixture was incubated in a plastic bag at room temperature in the dark until similar microbial abundance was observed among dilution treatments. Plastic bags were sealed using a sterilized cotton plug to avoid aerial microbial contamination while permitting gas exchange. **(3) Disturbances and incubation**. We used 50 ml conical Mini Bioreactors (Product Number 431720, Corning Inc., NY) as experimental units. Each Mini Bioreactor has four vents in the cap, where a hydrophobic membrane can avoid microbial contamination but allow gas exchange. Each Mini Bioreactor was filled with 5 g of soil inoculum and 35 g of sterilized soil, containing an appropriate dose of the chemical treatments. After implementing all global change factors, Mini Bioreactor containing soils were incubated for six weeks in the dark. **(4) Soil functions and properties**. We determined soil functions and properties,

including water-stability aggregates, water repellency, respiration, enzyme activity, decomposition rate, microbial abundance and biodiversity.

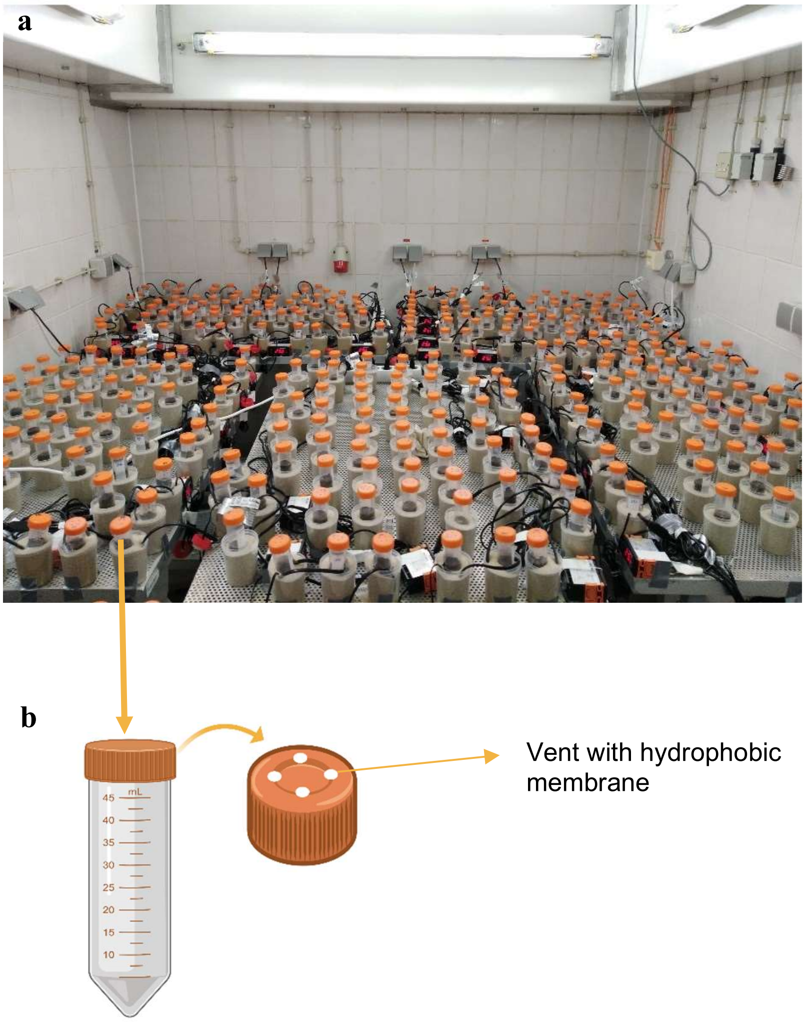

Supplementary Fig. 2 (**a**) A top view of the placement of experimental units in the climate chamber. The Mini Bioreactors with warming treatment were wrapped around with heating cables. A set temperature was maintained by temperature controllers, which can switch off and on heating cables depending on the real-time temperature of Mini Bioreactors' outside surface. Mini Bioreactors were placed in beakers filled with sand to reduce thermal radiation from neighboring units. (**b**) Four vents with a hydrophobic membrane in the cap. The hydrophobic membrane can avoid microbial contamination but allow gas exchange.

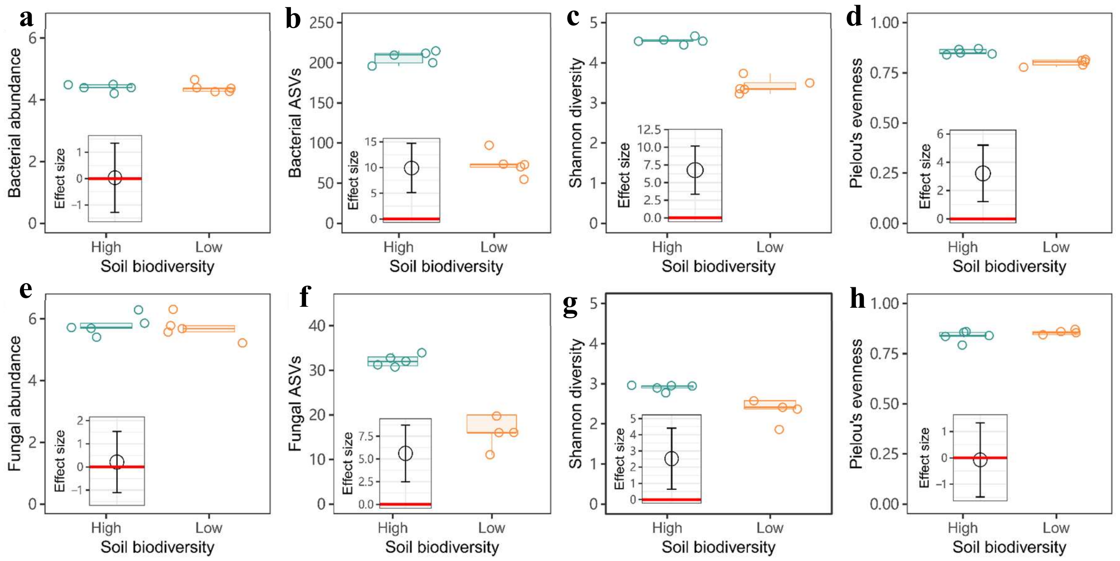

Supplementary Fig. 3 Effects of the dilution-to-extinction approach on the microbial abundance and diversity of soil inoculum after incubation (n = 5 for all panels). There were similar fungal (**a**) and bacterial (**e**) abundance (log-transformed copy number/g soil) in soil inoculum between the high and low soil biodiversity treatments, estimated by quantitative real-time PCR. The dilution-to-extinction approach dramatically decreased bacterial (**b**, **c** and **d**) and fungal (**f** and **g**) diversity indices. The upper and lower bounds of a boxplot are the 75th and 25th percentiles, respectively; the horizontal line inside the box is the median; the whiskers represent the 1.5 times interquartile range. For all panels (**a-h**), effect sizes (Hedges' g) were calculated as the difference of the means between high and low soil biodiversity treatments in units of the pooled standard deviation. Points are the mean values and the error bars are their 95% confidence intervals. The positive effect sizes without intervals overlapping zero (red line in subfigure of each figure) indicate that soil biodiversity increased soil functions and properties; the effect sizes with intervals overlapping zero are not significantly different from zero, meaning that soil biodiversity does not affect variables.

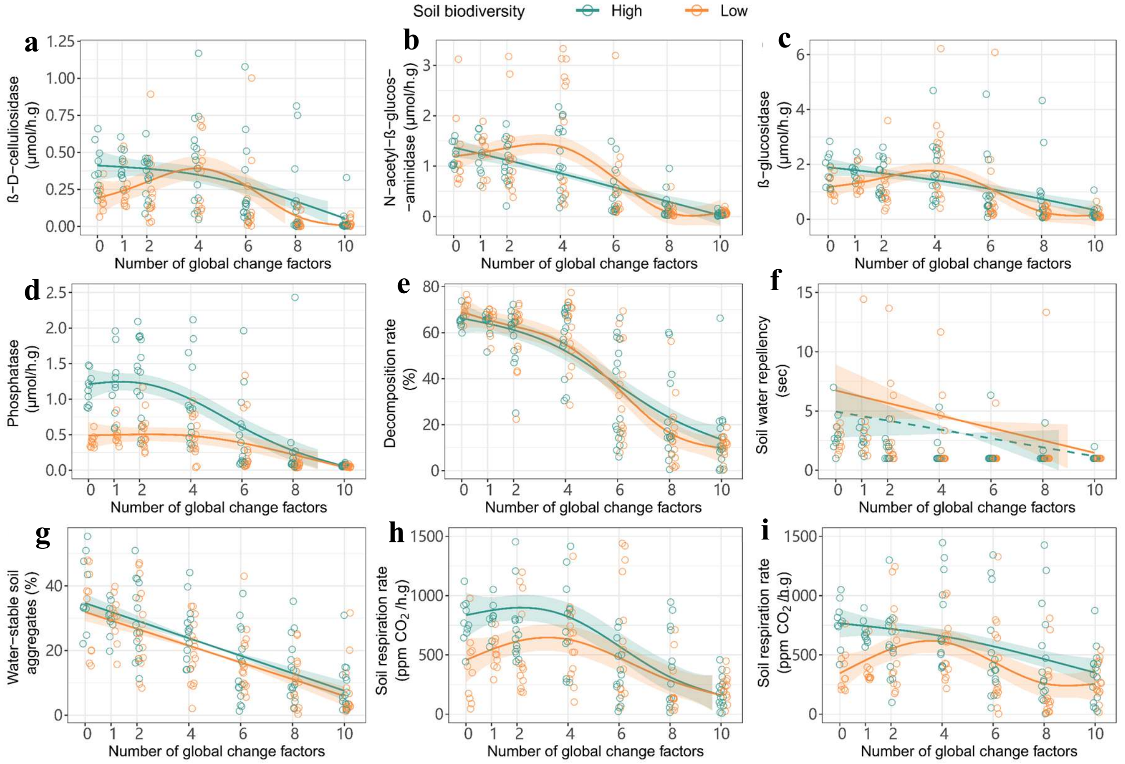

Supplementary Fig. 4 Effects of the number of global change factors (GCFs) on soil functions and properties in the high and low soil biodiversity treatments. **h** and **i** represent the soil respiration rates measured in the 3rd and last week, respectively. The observed values along with the number of GCFs with the fitted generalized additive models (mean and 95% confidence intervals). The significance of fitted models was indicated by solid (P < 0.05) and dashed (P > 0.05) lines (n = 95 for each line). The statistical test used was two-sided.

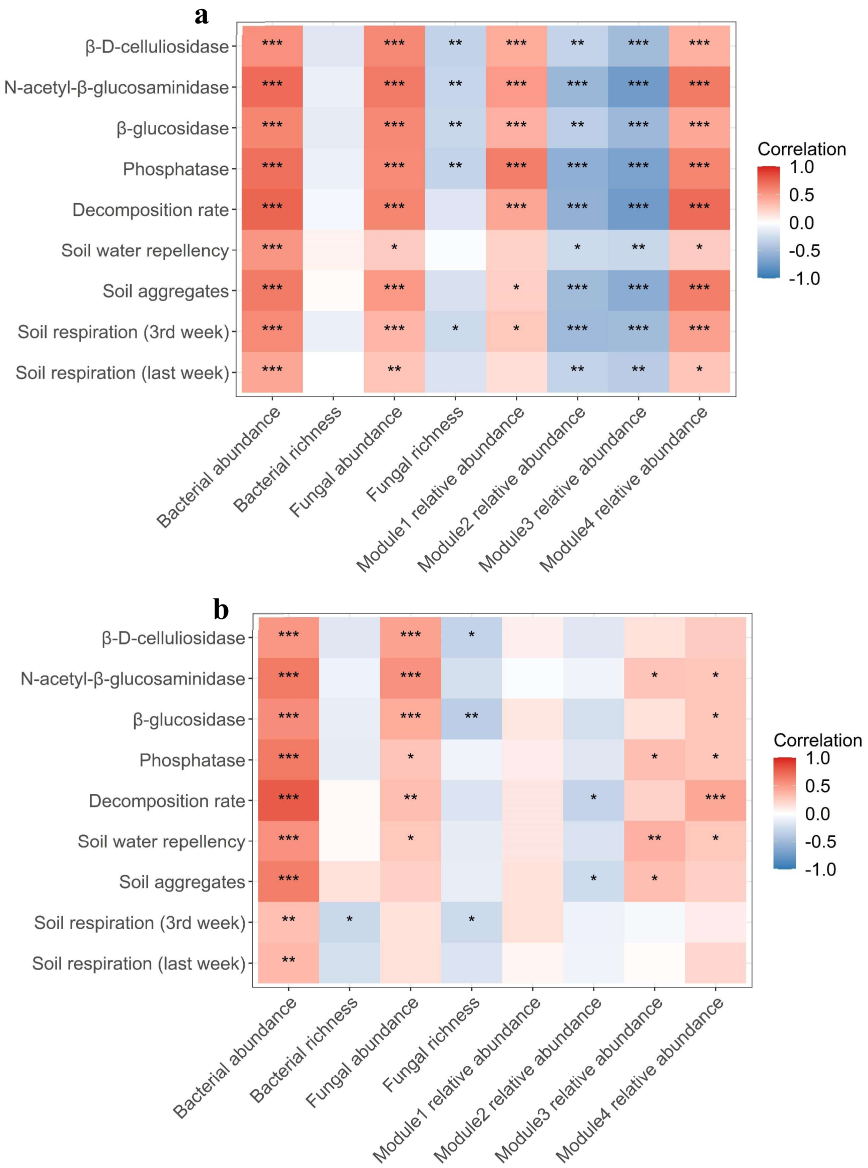

Supplementary Fig. 5 Relationships between soil microbial indices and soil functions and properties in the high (**a**) and low (**b**) soil biodiversity treatments. Spearman's rho statistic was employed to detect the statistical significance of Spearman correlations using a two-sided hypothesis test. The adjustment method "fdr" was used to control the false discovery rate for multiple testing correction. Positive and negative correlation coefficients are highlighted in red and blue, respectively. Asterisks represent the statistical significance of correlations ($n = 95$): *** $P < 0.001$, ** $P < 0.01$, * $P < 0.05$.

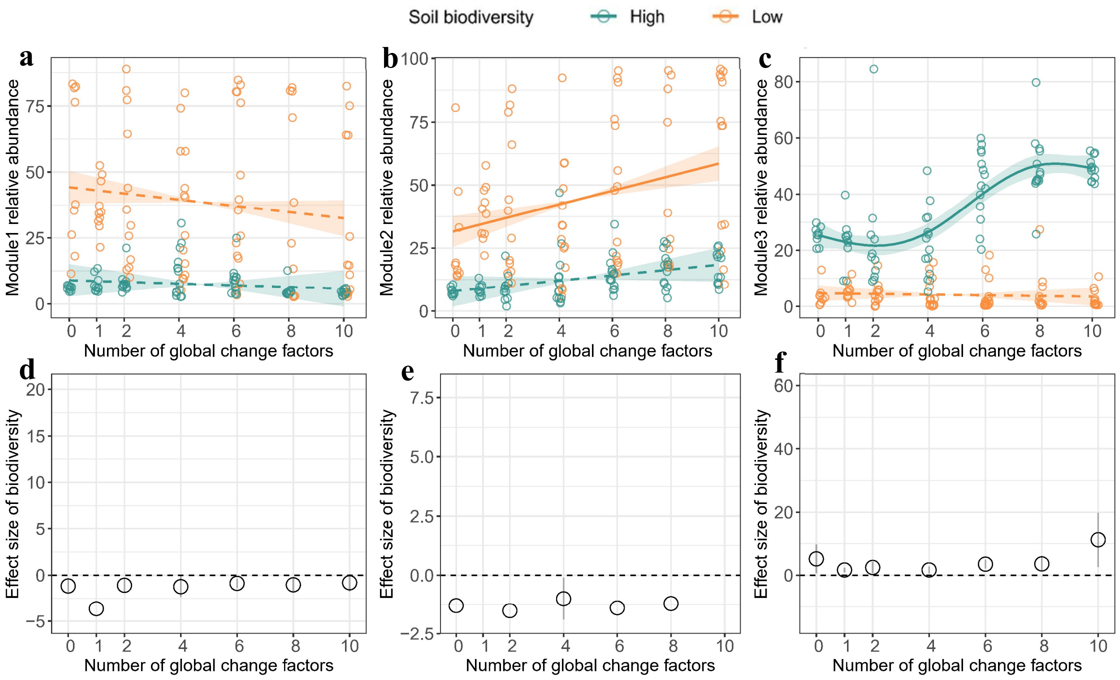

Supplementary Fig. 6 The effect of soil biodiversity and the increasing number of global change factors on the relative abundance of modules. (**a** – **c**) The effect of the increasing number of global change factors on the relative abundance of modules in high and low soil biodiversity treatments. The observed values along with the number of GCFs with the fitted generalized additive models (mean and 95% confidence intervals). The statistical significance of the fitted models was indicated by solid ($P < 0.05$) and dashed ($P > 0.05$) lines ($n = 95$ for each line). The statistical test used was two-sided. (**d** – **f**) The effect size of soil biodiversity on the relative abundance of modules along with an increasing number of global change factors. Effect sizes (Hedges' g) were calculated as the difference of the means between high and low soil biodiversity treatments in units of the pooled standard deviation. Points are the mean values and the error bars are their 95% confidence intervals ($n = 10$ for zero and single global change factors; $n = 15$ for multiple global change factors). The positive effect sizes without intervals overlapping zero indicate that soil biodiversity increased soil functions and properties; the effect sizes with intervals overlapping zero are not significantly different from zero, meaning that soil biodiversity does not affect variables.

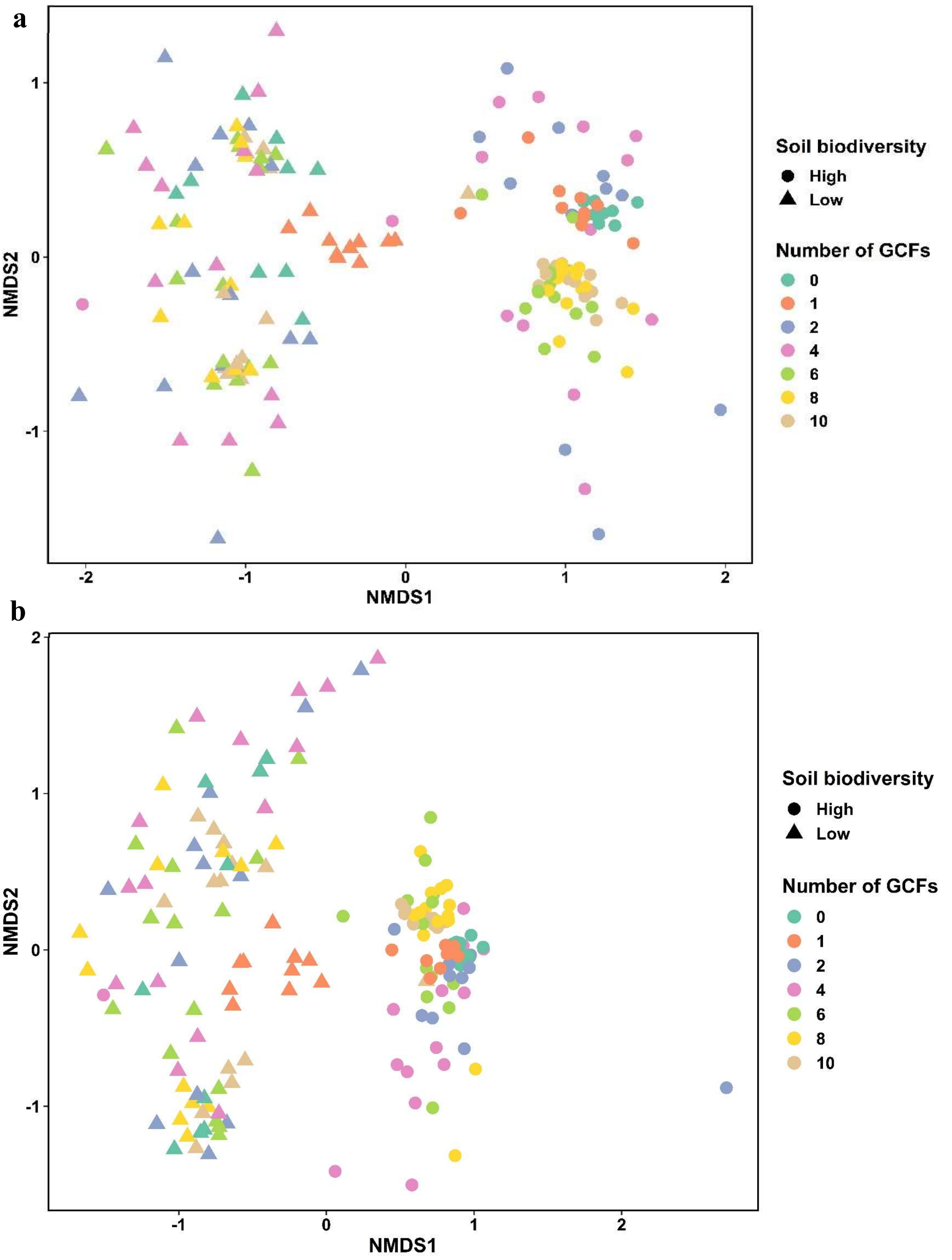

Supplementary Fig. 7 The dissimilarity of bacterial (**a**) and fungal (**b**) community composition among soil biodiversity and GCF treatments, summarized by the non-metric multidimensional scaling (NMDS) ordination based on Bray-Curtis distance.

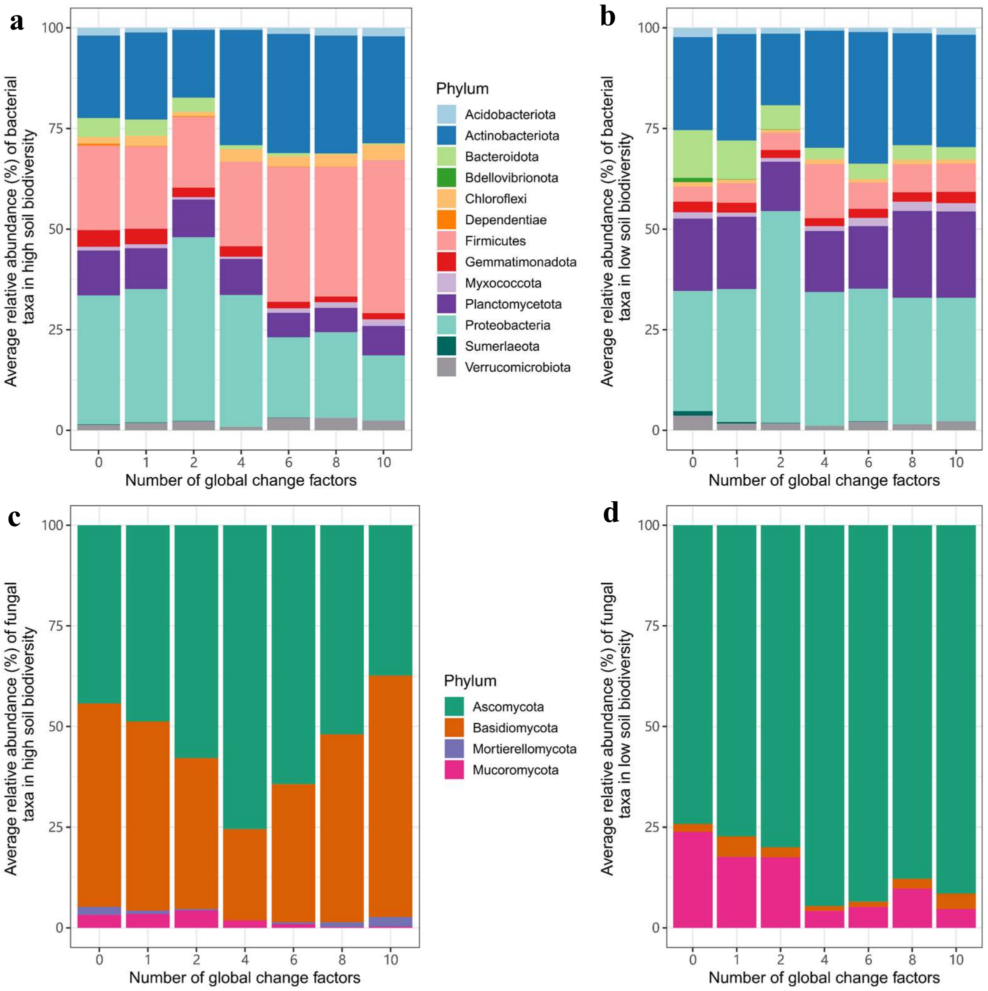

Supplementary Fig. 8 Effects of soil biodiversity and increasing number of global change factors (GCFs) on community composition of bacteria (**a** and **b**) and fungi (**c** and **d**). Only phyla with an appearance above 50% of replicates are plotted.

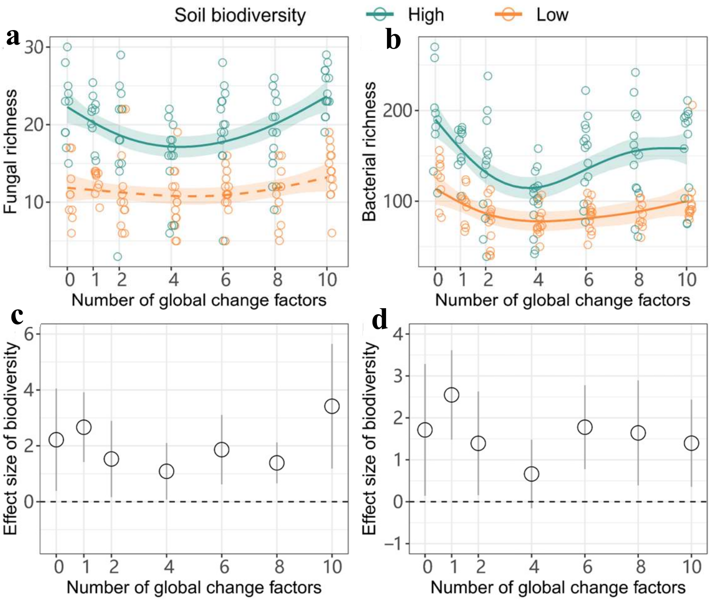

Supplementary Fig. 9 The effect of soil biodiversity and the increasing number of global change factors on bacterial and fungal richness (number of ASVs). (**a** – **b**) The effect of the increasing number of global change factors on bacterial and fungal richness in high and low soil biodiversity treatments. The observed values along with the number of GCFs with the fitted generalized additive models (mean and 95% confidence intervals). The significance of fitted models was indicated by solid ($P < 0.05$) and dashed ($P > 0.05$) lines ($n = 95$ for each line). The statistical test used was two-sided. (**c** – **d**) The effect size of soil biodiversity on bacterial and fungal richness along with an increasing number of global change factors. Effect sizes (Hedges' g) were calculated as the difference of the means between high and low soil biodiversity treatments in units of the pooled standard deviation. Points are the mean values and the error bars are their 95% confidence intervals ($n = 10$ for zero and single global change factors; $n = 15$ for multiple global change factors). The positive effect sizes without intervals overlapping zero indicate that soil biodiversity increased soil functions and properties; the effect sizes with intervals overlapping zero are not significantly different from zero, meaning that soil biodiversity does not affect variables.

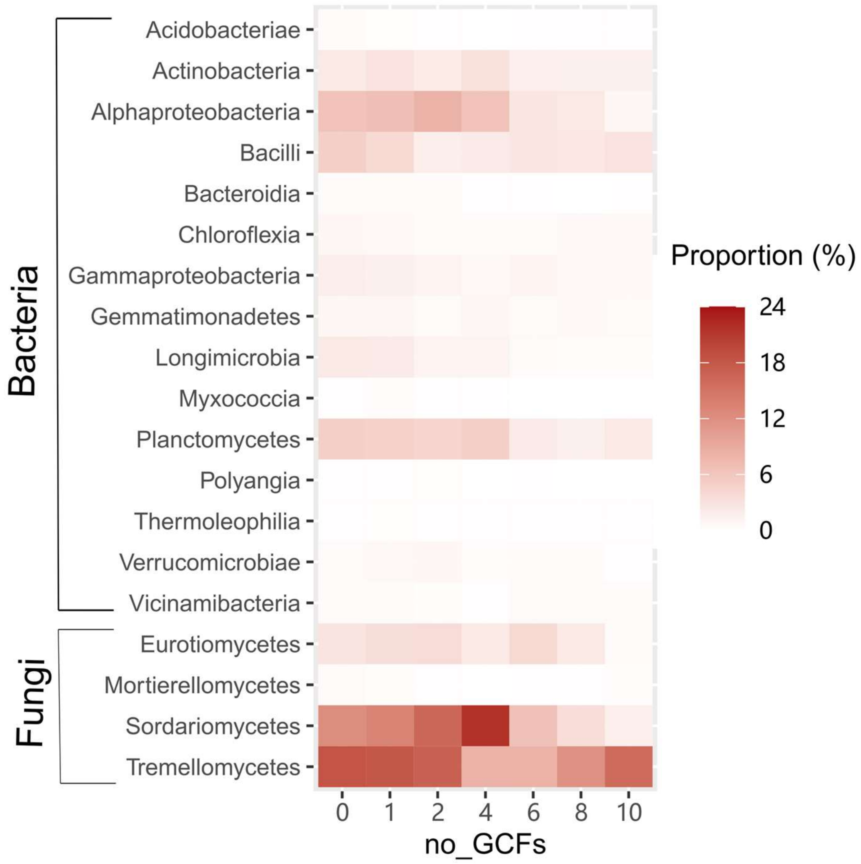

Supplementary Fig. 10 Effects of increasing number of global change factors (GCFs) on community composition of module 4.

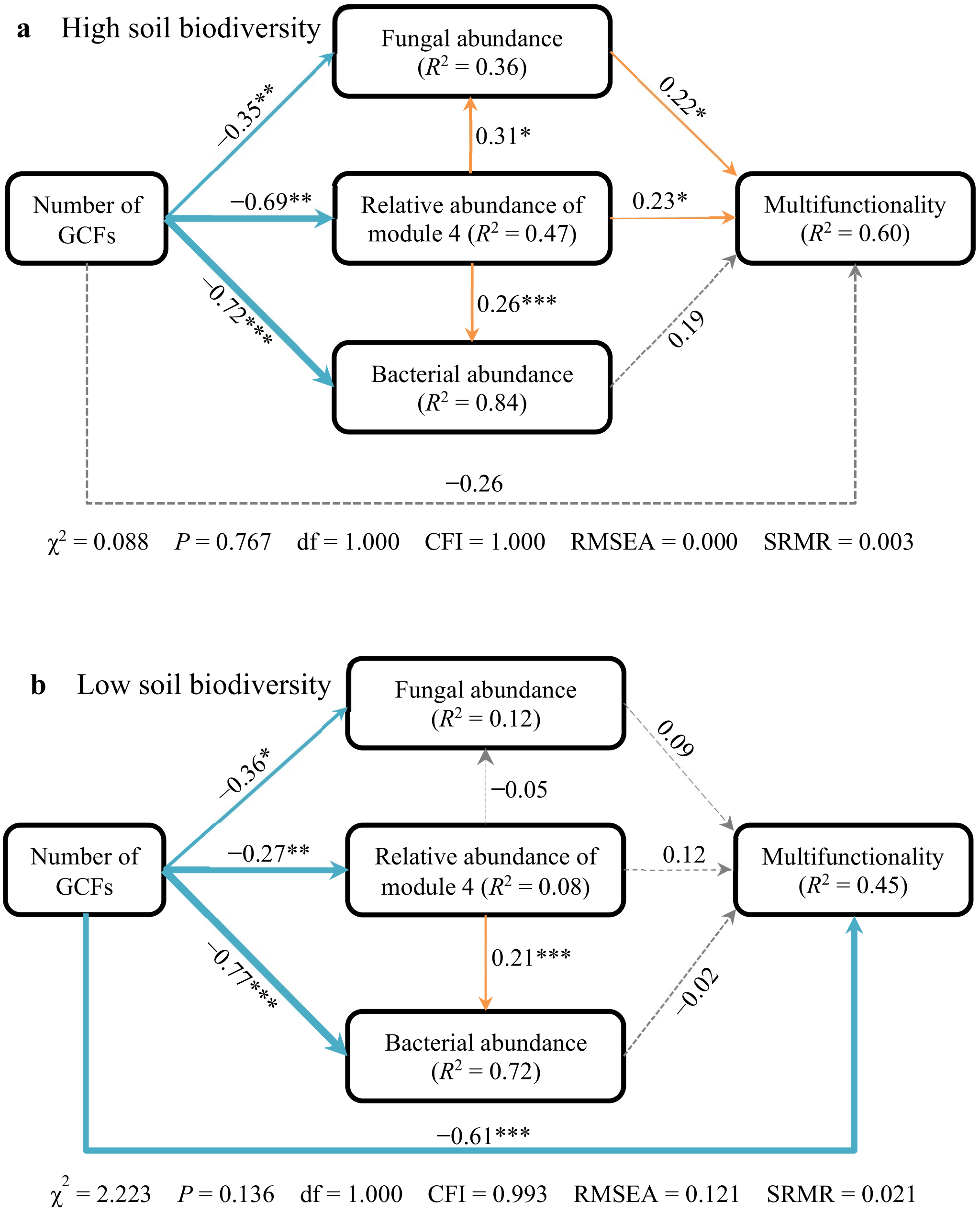

Supplementary Fig. 11 Structural equation models illustrating the direct and indirect effects of an increasing number of GCFs on soil multifunctionality in the high (**a**) and low (**b**) soil biodiversity treatments (n = 95). Solid orange arrows represent positive paths (P < 0.05), solid blue arrows represent negative paths (P < 0.05), and grey dashed arrows indicate non-significant paths (P > 0.05). Numbers adjacent to arrows are the standardized path coefficients. Arrow width is scaled to the magnitude of the standardized path

coefficients. R^2 indicates the proportion of variance explained by predictors. The goodness-of-fit statistics are shown at the bottom of each model: χ^2 and P values of test statistics (one sided), degrees of freedom, comparative fit index (CFI), root mean square error of approximation (RMSEA), and standardized root mean square residual (SRMR). Soil dilution has diminished module 4, and thus this module likely did not explain multifunctionality in the low-diversity treatment.

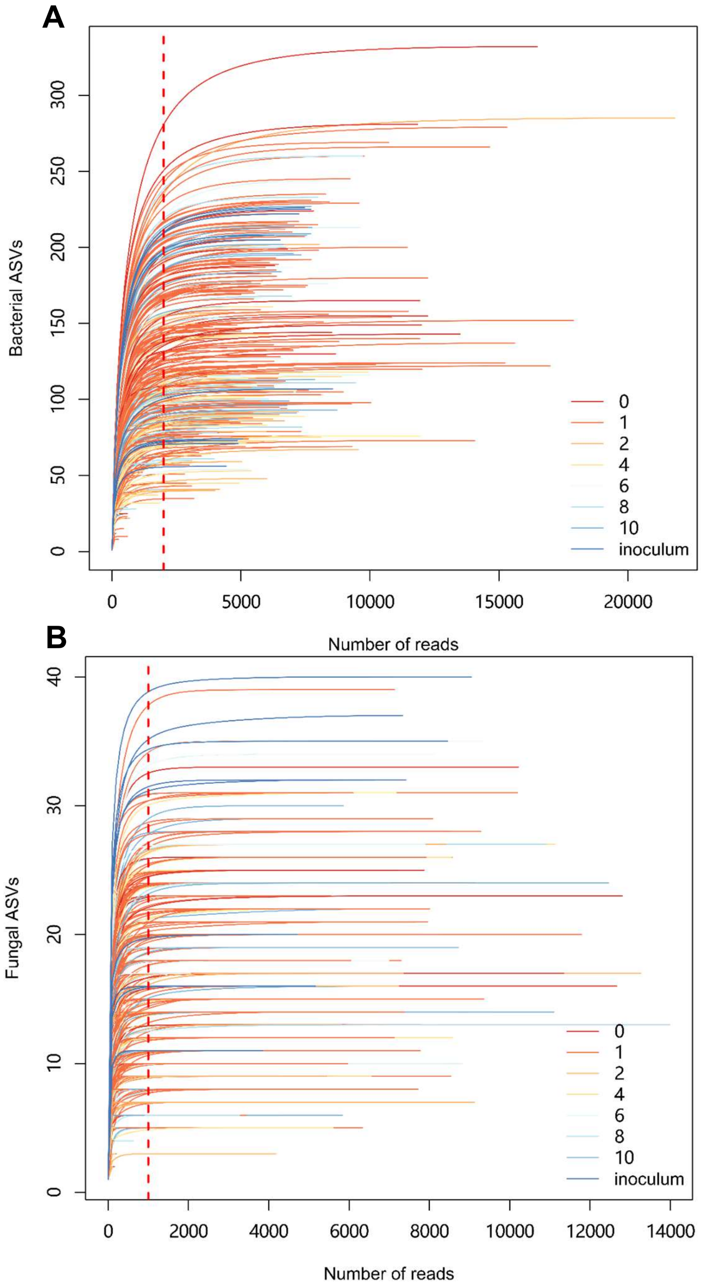

Supplementary Fig. 12 Rarefaction curves of bacterial (**A**) and fungal (**B**) gene reads for the sequenced samples from soil inoculum and soil of the last harvest.

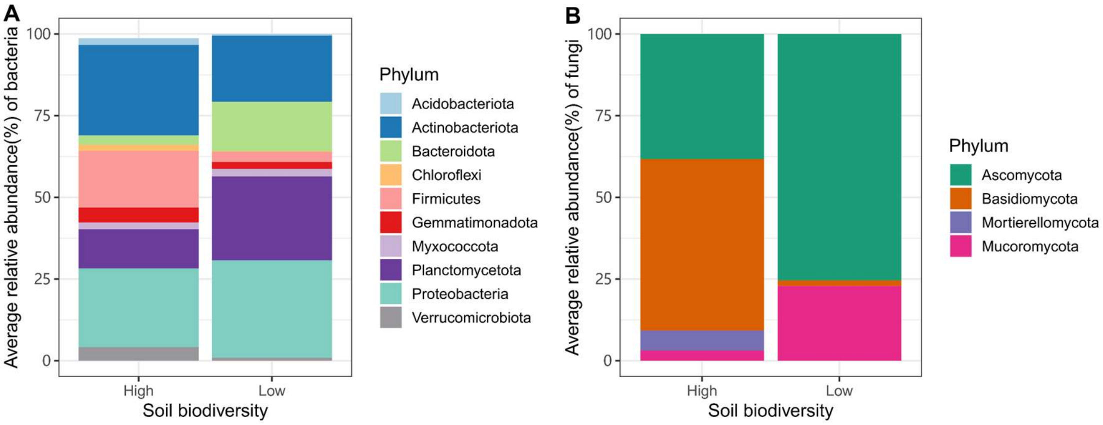

Supplementary Fig. 13 Community composition of bacteria (**A**) and fungi (**B**) in soil inoculum. Only phyla with a relative abundance above 5% are plotted.

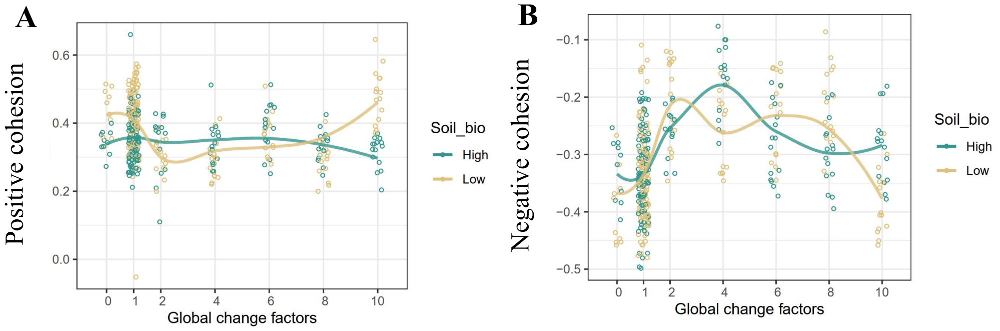

Supplementary Fig. 14 Positive and negative cohesions along an increasing number of GCFs within each soil biodiversity treatment.

References

1. Beck, M. E. & Wurst, S. Soil biota of different size classes change the impact of soil compaction on a plant community. *Appl Veg Sci* **16**, 650-657 (2013).
2. Roger, F., Bertilsson, S., Langenheder, S., Osman, O. A. & Gamfeldt, L. Effects of multiple dimensions of bacterial diversity on functioning, stability and multifunctionality. *Ecology* **97**, 2716-2728 (2016).
3. Yan, Y., Kuramae, E. E., Klinkhamer, P. G. L. & van Veen, J. A. Revisiting the dilution procedure used to manipulate microbial biodiversity in terrestrial systems. *Appl. Environ. Microbiol.* **81**, 4246-4252 (2015).
4. Maron, P. A. *et al.* High microbial diversity promotes soil ecosystem functioning. *Appl. Environ. Microbiol.* **84**, 9 (2018).
5. Domeignoz-Horta, L. A. *et al.* Microbial diversity drives carbon use efficiency in a model soil. *Nat. Commun.* **11**, 3684 (2020).
6. Hol, W. H. G. *et al.* Context dependency and saturating effects of loss of rare soil microbes on plant productivity. *Front. Plant Sci.* **6**, 485 (2015).
7. Gaston, K. Biodiversity and extinction: the importance of being common. *PPG: Earth and Environment* **32**, 73-79 (2008).
8. Zhou, Z., Wang, C. & Luo, Y. Meta-analysis of the impacts of global change factors on soil microbial diversity and functionality. *Nat. Commun.* **11**, 3072 (2020).
9. *Intergovernmental Panel on Climate Change (IPCC), "Climate change 2014: Synthesis report. A contribution of working groups I, II, and III to the fifth assessment report of the Intergovernmental Panel on Climate Change," Core Writing Team, R. K. Pachauri, L. A. Meyer, Eds. (IPCC, Geneva, Switzerland, 2014).*
10. Rillig, M. C. *et al.* The role of multiple global change factors in driving soil functions and microbial biodiversity. *Science* **366**, 886-890 (2019).
11. Vitousek, P. M. *et al.* Human alteration of the global nitrogen cycle: sources and consequences. *Ecol. Appl.* **7**, 737-750 (1997).

12. Lehner, B., Döll, P., Alcamo, J., Henrichs, T. & Kaspar, F. Estimating the impact of global change on flood and drought risks in Europe: a continental, integrated analysis. *Clim. Change* **75**, 273-299 (2006).
13. Markonis, Y. *et al.* The rise of compound warm-season droughts in Europe. *Sci Adv* **7**, eabb9668 (2021).
14. de Vries, F. T., Griffiths, R. I., Knight, C. G., Nicolitch, O. & Williams, A. Harnessing rhizosphere microbiomes for drought-resilient crop production. *Science* **368**, 270-274 (2020).
15. de Vries, F. T., Brown, C. & Stevens, C. J. Grassland species root response to drought: consequences for soil carbon and nitrogen availability. *Plant Soil* **409**, 297-312 (2016).
16. Yang, G., Roy, J., Veresoglou, S. D. & Rillig, M. C. Soil biodiversity enhances the persistence of legumes under climate change. *New Phytol.* **229**, 2945-2956 (2021).
17. Mishra, S. *et al.* in *Environmental Biotechnology: For Sustainable Future* (eds Ranbir Chander Sobti, Naveen Kumar Arora, & Richa Kothari) 103-125 (Springer Singapore, 2019).
18. Kumar Sharma, R., Agrawal, M. & Marshall, F. Heavy metal contamination of soil and vegetables in suburban areas of Varanasi, India. *Ecotoxicol. Environ. Saf.* **66**, 258-266 (2007).
19. Ballabio, C. *et al.* Copper distribution in European topsoils: an assessment based on LUCAS soil survey. *Sci. Total Environ.* **636**, 282-298 (2018).
20. He, H. *et al.* Distribution characteristics of residual film over a cotton field under long-term film mulching and drip irrigation in an oasis agroecosystem. *Soil Till Res* **180**, 194-203 (2018).
21. Qi, Y. *et al.* Effects of plastic mulch film residues on wheat rhizosphere and soil properties. *J. Hazard. Mater.* **387**, 121711 (2020).
22. Daliakopoulos, I. N. *et al.* The threat of soil salinity: a European scale review. *Sci. Total Environ.* **573**, 727-739 (2016).
23. Rengasamy, P. World salinization with emphasis on Australia. *J. Exp. Bot.* **57**, 1017-1023 (2006).

24. *Millennium Ecosystem Assessment, "Ecosystems and human well-being: Synthesis," (Island Press, Washington, DC, 2005).*
25. Hassani, A., Azapagic, A. & Shokri, N. Predicting long-term dynamics of soil salinity and sodicity on a global scale. *Proc. Natl. Acad. Sci. USA* **117**, 33017-33027 (2020).
26. Pelosi, C. *et al.* Residues of currently used pesticides in soils and earthworms: A silent threat? *Agric., Ecosyst. Environ.* **305**, 107167 (2021).
27. Riedo, J. *et al.* Widespread occurrence of pesticides in organically managed agricultural soils—the ghost of a conventional agricultural past? *Environ. Sci. Technol.* **55**, 2919-2928 (2021).
28. Chen, S.-K., Edwards, C. A. & Subler, S. Effects of the fungicides benomyl, captan and chlorothalonil on soil microbial activity and nitrogen dynamics in laboratory incubations. *Soil Biol. Biochem.* **33**, 1971-1980 (2001).
29. Rousk, J., Demoling, L. A. & Bååth, E. Contrasting short-term antibiotic effects on respiration and bacterial growth compromises the validity of the selective respiratory inhibition technique to distinguish fungi and bacteria. *Microb. Ecol.* **58**, 75-85 (2009).
30. Rousk, J., Demoling, L. A., Bahr, A. & Bååth, E. Examining the fungal and bacterial niche overlap using selective inhibitors in soil. *FEMS Microbiol. Ecol.* **63**, 350-358 (2008).
31. Bailey, V. L., Smith, J. L. & Bolton, H. Novel antibiotics as inhibitors for the selective respiratory inhibition method of measuring fungal:bacterial ratios in soil. *Biol. Fertility Soils* **38**, 154-160 (2003).
32. Jensen, J. Fate and effects of linear alkylbenzene sulphonates (LAS) in the terrestrial environment. *Sci. Total Environ.* **226**, 93-111 (1999).
33. Pandey, B. K. *et al.* Plant roots sense soil compaction through restricted ethylene diffusion. *Science* **371**, 276-280 (2021).
34. Bluett, C., Tullberg, J. N., McPhee, J. E. & Antille, D. L. Soil and tillage research: why still focus on soil compaction? *Soil Till Res* **194**, 2 (2019).

35. Devigne, C., Mouchon, P. & Vanhee, B. Impact of soil compaction on soil biodiversity - does it matter in urban context? *Urban Ecosyst.* **19**, 1163-1178 (2016).

36. Weyer, T. & Boeddinghaus, R. *Preventing soil compaction: preserving and restoring soil fertility: including the classification key for detection and evaluation of harmful soil compaction in the field*. (Ministry for Climate Protection, Environment, Agriculture, Conservation and Consumer Protection of the State North Rhine-Westphalia, 2016).

37. Lane, D. J. in *Nucleic acid techniques in bacterial systematics* (eds E. Stackebrandt & M. Goodfellow) 115-176 (John Wiley & Sons, Inc., 1991).

38. Fierer, N., Jackson, J. A., Vilgalys, R. & Jackson, R. B. Assessment of soil microbial community structure by use of taxon-specific quantitative PCR assays. *Appl. Environ. Microbiol.* **71**, 4117-4120 (2005).

39. Jackson, C. R., Tyler, H. L. & Millar, J. J. Determination of microbial extracellular enzyme activity in waters, soils, and sediments using high throughput microplate assays. *J. Vis. Exp.* **80**, e50399 (2013).

40. Delgado-Baquerizo, M. *et al.* Soil microbial communities drive the resistance of ecosystem multifunctionality to global change in drylands across the globe. *Ecol. Lett.* **20**, 1295-1305 (2017).

41. Kemper, W. D. & Rosenau, R. C. in *Methods of Soil Analysis. Part I – Physical and Mineralogical Methods* (ed A. Lute) 425–443 (Soil Science Society of America, 1986).

42. Hallett, P. D. in *Proceedings of the Eighth International Symposium on Adjuvants for Agrochemicals (ISAA)* (ed R. E. Gaskin) (International Society for Agrochemical Adjuvants, 2007).

43. Ihrmark, K. *et al.* New primers to amplify the fungal ITS2 region – evaluation by 454-sequencing of artificial and natural communities. *FEMS Microbiol. Ecol.* **82**, 666-677 (2012).

44. Ballhausen, M.-B., Hewitt, R. & Rillig, M. C. Mimicking climate warming effects on Alaskan soil microbial communities via gradual temperature increase. *Sci Rep-Uk* **10**, 8533 (2020).

45. Callahan, B. J. *et al.* DADA2: High-resolution sample inference from Illumina amplicon data. *Nat. Methods* **13**, 581 (2016).
46. *https://benjjneb.github.io/dada2/index.html*.
47. Martin, M. Cutadapt removes adapter sequences from high-throughput sequencing reads. *EMBnet.journal* **17**, 10-12 (2011).
48. Wang, Q., Garrity, G. M., Tiedje, J. M. & Cole, J. R. Naive Bayesian classifier for rapid assignment of rRNA sequences into the new bacterial taxonomy. *Appl. Environ. Microbiol.* **73**, 5261-5267 (2007).
49. Nilsson, R. H. *et al.* The UNITE database for molecular identification of fungi: handling dark taxa and parallel taxonomic classifications. *Nucleic Acids Res.* **47**, D259-D264 (2019).
50. Yilmaz, P. *et al.* The SILVA and "All-species Living Tree Project (LTP)" taxonomic frameworks. *Nucleic Acids Res.* **42**, D643-D648 (2014).
51. Quast, C. *et al.* The SILVA ribosomal RNA gene database project: improved data processing and web-based tools. *Nucleic Acids Res.* **41**, D590-D596 (2013).
52. Herren, C. M. & McMahon, K. D. Cohesion: a method for quantifying the connectivity of microbial communities. *ISME J.* **11**, 2426-2438 (2017).
53. Hernandez, D. J., David, A. S., Menges, E. S., Searcy, C. A. & Afkhami, M. E. Environmental stress destabilizes microbial networks. *ISME J.* (2021).
54. Jing, X. *et al.* Variation in the methods leads to variation in the interpretation of biodiversity-ecosystem multifunctionality relationships. *Journal of Plant Ecology* **13**, 431–441 (2020).
55. Byrnes, J. E. K. *et al.* Investigating the relationship between biodiversity and ecosystem multifunctionality: challenges and solutions. *Methods Ecol. Evol.* **5**, 111-124 (2014).
56. Manning, P. *et al.* Redefining ecosystem multifunctionality. *Nat. Ecol. Evol.* **2**, 427-436 (2018).
57. Kassambara, A. & Mundt, F. *factoextra*: Extract and visualize the results of multivariate data analyses. *R* package version 1.0.7. https://CRAN.R-project.org/package=factoextra. (2020).
