# Supplementary material for: Multiple anthropogenic pressures eliminate the effects of soil microbial diversity on ecosystem functions in experimental microcosms
Source: Nat Commun. 2022 Jul 23;13:4260. doi: 10.1038/s41467-022-31936-7 (PMC9308766; doi:10.1038/s41467-022-31936-7)
Supplement: Supplementary file 4 — Reporting Summary [file 41467_2022_31936_MOESM4_ESM.pdf]

nature portfolio

Corresponding author(s): Gaowen Yang

Last updated by author(s): Jul 1, 2022

Reporting Summary

Nature Portfolio wishes to improve the reproducibility of the work that we publish. This form provides structure for consistency and transparency in reporting. For further information on Nature Portfolio policies, see our Editorial Policies and the Editorial Policy Checklist.

Statistics

For all statistical analyses, confirm that the following items are present in the figure legend, table legend, main text, or Methods section.

n/a	Confirmed	
☐	☒	The exact sample size (*n*) for each experimental group/condition, given as a discrete number and unit of measurement
☐	☒	A statement on whether measurements were taken from distinct samples or whether the same sample was measured repeatedly
☐	☒	The statistical test(s) used AND whether they are one- or two-sided<br>*Only common tests should be described solely by name; describe more complex techniques in the Methods section.*
☒	☐	A description of all covariates tested
☐	☒	A description of any assumptions or corrections, such as tests of normality and adjustment for multiple comparisons
☐	☒	A full description of the statistical parameters including central tendency (e.g. means) or other basic estimates (e.g. regression coefficient) AND variation (e.g. standard deviation) or associated estimates of uncertainty (e.g. confidence intervals)
☐	☒	For null hypothesis testing, the test statistic (e.g. *F*, *t*, *r*) with confidence intervals, effect sizes, degrees of freedom and *P* value noted<br>*Give P values as exact values whenever suitable.*
☒	☐	For Bayesian analysis, information on the choice of priors and Markov chain Monte Carlo settings
☒	☐	For hierarchical and complex designs, identification of the appropriate level for tests and full reporting of outcomes
☐	☒	Estimates of effect sizes (e.g. Cohen's *d*, Pearson's *r*), indicating how they were calculated

Our web collection on statistics for biologists contains articles on many of the points above.

Software and code

Policy information about availability of computer code

Data collection	No special software was used to collect data.
Data analysis	We used the package FUNGuildR(R package version 0.2.0.9000) to taxonomically parse fungal trait information. All data analyses were performed in R for Windos 11 version 4.1.3 (www.R-project.org). The R script is available in a publicly accessible database (https://doi.org/10.6084/m9.figshare.16988539.v6).

For manuscripts utilizing custom algorithms or software that are central to the research but not yet described in published literature, software must be made available to editors and reviewers. We strongly encourage code deposition in a community repository (e.g. GitHub). See the Nature Portfolio guidelines for submitting code & software for further information.

Data

Policy information about availability of data

All manuscripts must include a data availability statement. This statement should provide the following information, where applicable:

- Accession codes, unique identifiers, or web links for publicly available datasets
- A description of any restrictions on data availability
- For clinical datasets or third party data, please ensure that the statement adheres to our policy

All datasets that support the findings of this study have been deposited in the figshare: https://doi.org/10.6084/m9.figshare.16988539.v6. The Silva 138.1 database used for bacteria taxonomic annotation is available in the zenodo: https://zenodo.org/record/4587955#.YrZyNshfh_8. For fungal taxonomic annotation, the UNITE ITS database is available in the webpage: https://unite.ut.ee/repository.php. The FUNGuild database is available in the webpage: http://www.stbates.org/funguild_db_2.php.

Field-specific reporting

Please select the one below that is the best fit for your research. If you are not sure, read the appropriate sections before making your selection.

☐ Life sciences ☐ Behavioural & social sciences ☒ Ecological, evolutionary & environmental sciences

For a reference copy of the document with all sections, see nature.com/documents/nr-reporting-summary-flat.pdf

Ecological, evolutionary & environmental sciences study design

All studies must disclose on these points even when the disclosure is negative.

Study description	This experiment was set up as a fully factorial design containing all combinations of two levels of soil biodiversity (high and low) and seven levels of the number of global change factors (GCFs) (0, 1, 2, 4, 6, 8, 10 GCFs). There were 10 replicates for the control (zero GCF treatment), 10 for each GCF at single GCF treatment, and 15 replicates for each combined GCF level, for a total of 370 experimental units. The combined GCF treatments were created by randomly selecting GCF from a pool of 10 CGFs. For each replicate of combined GCF levels, there were identical CGF combinations between the high and low soil biodiversity treatments to avoid a confounding effect of CGF combination and soil biodiversity treatments.
Research sample	Soils were collected from the top 10 cm of a farmland in Albrecht-Thaer-Weg, Berlin (52.466°N, 13.303°E). The co-occurrence of multiple pressures, or at least some combination of pressures, including nutrient eutrophication, warming, drought, mechanic compaction, heavy metal pollution, residues of plastic mulching film and pesticides, has been reported by recent studies in intensively agroecosystems. Therefore, we used the field soil of a farmland to test the effect of soil microbial diversity under the co-occurrence of multiple pressures.
Sampling strategy	Sample size was determined based on our previous study with similar experimental design and materials. In this previous study (Rillig et al 2019, Science, 366, 886-890), we had the following levels of replication: control (n=20), individual factors (n=8 each), and factor richness levels (n=10 each). We found that there was much lower standard deviations among repeats in the control, compared those in the individual factor and factor richness treatments in this previous study. Thus, in the present study, the replication of control was reduced to 10, while repeats of individual factor and factor richness treatments were promoted to 10 and 15, respectively.
Data collection	We measured the following response variables: soil respiration determined by an infrared gas analyzer (LiCOR 6400xt, Lincoln, NE, USA), bacterial and fungal abundance estimated by quantitative real-time PCR (CFX Real-Time System, C1000 Touch™ Thermal Cycler, BIO-RAD, California, USA), litter decomposition rate tested by a balance, soil enzyme activity measured using a microplate reader (BioRad, Benchmark Plus, Japan), water-stable soil aggregates tested using a sieving machine (Agrisearch Equipment, Eijkelkamp, Giesbeek, Netherlands) and soil water repellency measured by a chronometer, the taxonomic composition of soil fungal and bacterial communities determined by the high throughput sequencing (Illumina MiSeq). Gaowen Yang colleted and recorded data using Excel, with the help from Daniel R. Lammel and Max-Bernhard Ballhausen.
Timing and spatial scale	This experiment started on 17/08/2022 and stopped on 30/09/2020. In our previous study (Rillig et al 2019), six weeks were enough to detect significant responses of soil properties and functions to multiple GCF treatments. Therefore, soils with treatments were incubated for six weeks, and then we sampled all soils and did the measurement in the present study. Soil respirations were measured in the 3rd and 6th week before harvest to capture the response of microbial activity across time scale, and similar response trend were observed in the 3rd and 6th week. Spatial scale was not considered in this study, because all 40 g of soil in each experimental unit was used for measurements.
Data exclusions	No data were excluded from the analyses, with the exception of microbial analysis. For diversity and community composition analysis, we excluded the samples with less than 1% of the observations of the largest sample in the ASV table. For network analysis, we then removed ASVs with low prevalence, which presented less than 20% of samples across all experimental units to reduce the high percentage of zero counts. Isolated nodes was removed before network visualization.
Reproducibility	There were a large number of replicates, e.g., 15 repeats for the multiple GCF treatments, and 7 gradients of GCF treatments, which lead to a total of 370 experimental units. Furthermore, all experimental units were incubated in a climate chamber with a precise control of temperature. These will ensure the confidence that the detected response trend could be generalized.
Randomization	All treatments were randomly allocated to each experimental unit (Mini Bioreactor). Besides, all Mini Bioreactors were randomly located in the incubation room.
Blinding	Each sample was labeled by a number during sampling and measurement. The number did not indicate a treatment of a sample.

Did the study involve field work? ☒ Yes ☐ No

Field work, collection and transport

Field conditions	We collected field soil from the top 10 cm of a farmland in Albrecht-Thaer-Weg, Berlin (52.466°N, 13.303°E). The soil is an Albic Luvisol and has the following properties: 73.6% sand, 18.8% silt and 7.6% clay; pH 7.1 (CaCl2), 6.9 mg P/100 g soil (calciumacetate-lactate), 0.12% nitrogen and 1.87% carbon. The weather conditions are available form a nearby meteorological station (https://www.agrar.hu-berlin.de/de/institut/departments/dntw/agrarmet/service/wo/Klima-Dahlem.html). Annual mean air temperature for 1991-2020 is 10.4 °C. Mean soil temperature in the growing season from May to October in the 0-10 cm is 20 °C, which was used as the incubation temperature in this experiment. Mean annual precipitation for 1991-2020 is 561.6 mm.

Location	A farmland in Albrecht-Thaer-Weg, Berlin (52.466°N, 13.303°E)
Access & import/export	A small number of soils (16 kg) were collected from the experimental field site of the Freie Universität Berlin, and no permit is required.
Disturbance	The collection of 16 kg of soils on a farmland had minor effects on agroecosystem.

Reporting for specific materials, systems and methods

We require information from authors about some types of materials, experimental systems and methods used in many studies. Here, indicate whether each material, system or method listed is relevant to your study. If you are not sure if a list item applies to your research, read the appropriate section before selecting a response.

Materials & experimental systems

n/a	Involved in the study
☒	☐ Antibodies
☒	☐ Eukaryotic cell lines
☒	☐ Palaeontology and archaeology
☒	☐ Animals and other organisms
☒	☐ Human research participants
☒	☐ Clinical data
☒	☐ Dual use research of concern

Methods

n/a	Involved in the study
☒	☐ ChIP-seq
☒	☐ Flow cytometry
☒	☐ MRI-based neuroimaging
